# Comparative Analysis of Viscoelastic Properties of Open Graded Friction Course under Dynamic and Static Loads

**DOI:** 10.3390/polym13081250

**Published:** 2021-04-12

**Authors:** Liding Li, Chunli Wu, Yongchun Cheng, Yongming Ai, He Li, Xiaoshu Tan

**Affiliations:** College of Transportation, Jilin University, Changchun 130025, China; lild17@mails.jlu.edu.cn (L.L.); clwu@jlu.edu.cn (C.W.); chengyc@jlu.edu.cn (Y.C.); lihe326532558@163.com (H.L.); tanxs20@mails.jlu.edu.cn (X.T.)

**Keywords:** road engineering, open graded friction course, viscoelastic properties, creep compliance, relaxation modulus, dynamic modulus, linear correlation

## Abstract

The viscoelastic properties of open graded friction course (OGFC) are closely related to anti-permanent deformation ability, noise reduction ability and durability. To study the viscoelastic parameters of OGFC under dynamic and static loads and to establish the functional relationship between them, uniaxial compression creep tests and dynamic modulus tests were performed to obtain the creep compliance and the dynamic modulus of OGFC. In addition, the Burgers model, modified Burgers model, second-order extensive Maxwell model, Scott-Blair model and modified Sigmoid model were employed to quantitatively analyze the dynamic and static viscoelastic properties of OGFC. Subsequently, the relaxation modulus of OGFC was deduced by the viscoelastic theory. Then, the dynamic modulus of OGFC was calculated according to the deduced relaxation modulus. Based on the calculated values and the measured values of dynamic modulus, the functional relationship of viscoelastic parameters of OGFC under dynamic and static loads was established. The results show that the increase in test temperature has adverse effects on the viscoelastic indexes of OGFC, such as creep compliance, relaxation modulus, and dynamic modulus; the dynamic modulus derived from static creep compliance has a good linear correlation with that obtained by dynamic modulus tests, but the correlation of the phase angle is poor.

## 1. Introduction

Open-graded friction course (OGFC) is a kind of open-graded asphalt mixture composed of aggregate and high viscosity modified asphalt. It has been widely used in permeable pavement engineering because of its many advantages, such as high permeability, skid resistance, and noise reduction [1,2]. Compared with the traditional dense-graded asphalt mixtures, the coarse-aggregate fraction of OGFC is mainly stone-on-stone contact, and its mechanical properties are relatively weak [1]. To improve the mechanical properties and durability of OGFC, some high performance modifiers (such as styrene-butadiene-styrene [3], rubber powder [4], polyvinyl chloride [5], polyurethane [1], and so on) and reinforcing fibers (such as steel fiber [5], glass fiber [6], basalt fiber [7], lignin fiber [8], acrylic fiber [7], and so on) are often used in OGFC. Moreover, to increase the cohesive bonding between the stone-on-stone contacts, the asphalt film thickness of the wrapped aggregate is increased, which makes OGFC have better viscoelastic deformation characteristics.

Relevant studies have pointed out that the viscoelastic parameters, such as creep compliance, relaxation modulus, dynamic modulus, and phase angle, are closely related to the anti-permanent deformation ability, noise reduction ability, and durability of OGFC. The creep compliance and relaxation modulus of OGFC can be obtained by the static uniaxial compression tests; and the dynamic modulus and phase angle used to describe the resistance to dynamic load deformation and to characterize the rheological properties of asphalt mixture can be tested through dynamic uniaxial compression tests. Biligiri et al. measured the phase angle of OGFC based on the dynamic modulus tests and analyzed the correlation between the phase angle of OGFC and pavement noise level [9]. Some research on the viscoelastic properties of OGFC can also provide a theoretical basis for sustainable flexible pavement design [10]. The relevant viscoelastic test or viscoelastic theory is also widely used in the mix proportion design and mechanical properties evaluation of OGFC. Pattanaik et al. used static creep tests and dynamic creep tests to study the optimum content of steel slag for OGFC [11]. Yi et al. analyzed the damage mechanism of OGFC under freeze-thaw cycle by using the viscoelastic-plastic damage model constructed by the generalized Maxwell model and the Drucker-Prager model [12]. Sarkar et al. analyzed the anti-rutting characteristics of OGFC by dynamic creep tests [6]. Hafeez et al. evaluated the influence of load waveform and load pulse duration on the permanent deformation resistance of OGFC [13]. The results showed that the pulse width and the type of axle related to the waveform had significant influence on the permanent deformation of asphalt mixtures. It can be seen from the above literature that the viscoelastic parameters are widely used to evaluate the road performance of OGFC, which means that the study of viscoelastic parameters of OGFC has great practical engineering significance.

At present, many researchers are also committed to using meso-mechanical models and mathematical algorithms to predict viscoelastic parameters (such as dynamic modulus and phase angle of OGFC), which can provide a good help for the study of viscoelastic properties of OGFC. Naik et al. and Venudharan et al. used robust mathematical functions and operators in the form of beta distribution to predict the elastic modulus and phase angle parameters of OGFC [4,14]. The results showed that there was a good correlation between the predicted values and the measured values. Zhang et al. predicted the dynamic modulus and phase angle of OGFC using a meso-mechanical model, but the results showed that the Dilute model, Mori-Tanaka model, Lielens′ model and generalized self-consistent model obtained lower dynamic modulus and higher phase angle, while the self-consistent model had higher dynamic modulus and smaller phase angle [15]. The above results show that the correlation of the prediction results of the meso-mechanical model is weak, and the prediction of the mathematical algorithm needs to accumulate a large number of experimental data to train the mathematical model.

However, the related studies have pointed out that there is an inherent relationship between dynamic modulus parameters and static creep compliance parameters of asphalt mixtures, and the creep compliance in the time domain can be transformed into dynamic modulus in the frequency domain according to the relevant mathematical theories [16,17]. Furthermore, the relationship between the creep compliance, relaxation modulus and dynamic modulus of asphalt mixtures can be established, so as to realize the prediction of dynamic modulus parameters. In addition, the static creep compliance can be obtained by static uniaxial compression creep test, and the test process is relatively simple and easy [18,19].

Therefore, to establish the functional relationship of viscoelastic parameters of OGFC under dynamic and static loads and realize the rapid prediction of dynamic parameters of OGFC, the creep compliance and the dynamic modulus of OGFC is tested by uniaxial compression creep tests and dynamic modulus tests at different temperatures. In addition, the relaxation modulus data of OGFC is needed to construct this function, but there is still a lack of effective testing equipment for testing the relaxation modulus. Thus, according to the viscoelastic theory, the relaxation modulus of OGFC is deduced by the convolution integral and Simpson quadrature formula. Then, the dynamic modulus parameters of OGFC are calculated and compared with the measured results. Based on the calculated values and the measured values, the functional relationship of viscoelastic parameters of OGFC under dynamic and static loads is established to realize the prediction of the dynamic modulus parameters from static creep compliance.

## 2. Materials and Methods

### 2.1. Materials and Sample Preparation

In this study, rubber modified asphalt was applied as the binder to prepare the open graded asphalt mixtures. The physical performance parameters of rubber modified asphalt was tested and is shown in Table 1. The performance of rubber modified asphalt is better than that of A-90 # base asphalt (unmodified asphalt). Basalt with a nominal maximum aggregate size of 13.2 mm was used as the mineral aggregate, and the gradation adopted in the study was shown in the Table 2. The aggregate with a particle size of less than 0.075 mm is limestone. The common properties of coarse and fine aggregates were tested and meet the specification requirements. Asphalt mixture samples (ϕ 150 mm × 180 mm) with 5.0% asphalt—aggregate ratio were fabricated by the Superpave gyratory compactor (Pine Instrument Company, PA Grove City, USA), and the test samples (ϕ 100 mm × 150 mm) were obtained by the core drilling method and cutting method.

### 2.2. Experimental Methods

#### 2.2.1. Uniaxial Compression Creep Test

To evaluate the viscoelastic properties of asphalt mixtures under static load, uniaxial compression creep tests at 10 °C, 20 °C, 30 °C, 40 °C, and 50 °C were carried out for three specimens in each group. For the static creep tests, the asphalt mixture specimen should be kept at the corresponding test temperature for 4 h before the test, so that the internal and external temperature of the specimen are uniform and reach the value required by the test. During the test, the contact pressure of 0.2 MPa was implemented on the cylindrical specimen, which was continued for 3600 s in the environment box at the corresponding test temperature [20,21]. All the creep tests were carried out on Cooper tester produced by Cooper Research Technology Ltd., Ripley, UK. In order to eliminate the slight unevenness on the surface of the specimen, it was necessary to prepress for 30 s before the formal test, with 0.015 MPa of pressure applied, and then the test load of 0.2 MPa was suddenly applied to the specimen for 3600 s. The creep compliances J(t) of OGFC can be calculated and obtained by Equation (1):

(1) $$J(t)=\frac{\varepsilon (t)}{\sigma_{0}}=\frac{U(t)}{h\sigma_{0}}$$

where U(t) is the variation of creep deformation for OGFC with loading time; h is the height of specimens for effective creep deformation; ε(t) is the response for creep strain, ε(t)=U(t)/h; $\sigma_{0}$ is the loading stress, $\sigma_{0}$ = 0.2 MPa. 

#### 2.2.2. Dynamic Modulus Test

To analyze the viscoelastic properties of OGFC under dynamic loads and compare the viscoelastic parameters between dynamic and static loads, dynamic modulus tests at 10 °C, 20 °C and 30 °C were performed for three specimens in each group according to the specification JTG E20-2011. The stress control was adopted throughout the tests, the loading amplitude was set to 0.2 MPa, and the loading frequencies were 0.1 Hz, 0.5 Hz, 1 Hz, 5 Hz, 10 Hz and 25 Hz, respectively [22,23]. Before the test, the asphalt mixtures specimens were kept at the corresponding test temperature for 4 h. The dynamic modulus G* and phase angle δ can be obtained by Equations (2) and (3):

(2) $$G*=\frac{\sigma_{i}}{\varepsilon_{i}}$$

(3) $$\delta =\frac{t_{i}}{t_{p}}\times 360$$

where $\sigma_{i}$ is the average amplitude of axial compressive stress for the last five loading cycles; $\varepsilon_{i}$ is the average amplitude of axial strain for the last five loading cycles; $t_{i}$ is the average delay time between the peak value of strain and the peak value of stress in the last five loading cycles; $t_{p}$ is the average loading period in the last five loading cycles. 

#### 2.2.3. Interconversion between Relaxation Modulus and Creep Compliance

Creep and relaxation are two kinds of mechanical responses of viscoelastic materials, such as asphalt mixtures, under different loading modes. Some studies have pointed out that there is a function relationship between the creep compliance and the relaxation modulus as shown in Equation (1) [17,18,24,25].

(4) $$\int_{0}^{t_{n}}G(\xi )J(t-\xi )d\xi =t_{n}$$

where *E*(*ξ*) is the relaxation modulus of OGFC at loading time *ξ*, and *J*(*t − ξ*) is the creep compliance of OGFC at loading time t − ξ. According to the principle of integral superposition, Equation (4) can be divided into Equation (5):

(5) $$\int_{0}^{t_{n}}G(\xi )J(t_{n}-\xi )d\xi =\sum_{i=1}^{n}\int_{t_{i-1}}^{t_{i}}G(\xi )J(t_{n}-\xi )d\xi =t_{n}$$

According to the numerical integration algorithm and the Simpson rule summary, the relaxation modulus of OGFC can be calculated. The calculation method is shown in Equation (6):

(6) $$\sum_{i=1}^{n}G(\frac{t_{i-1}+t_{i}}{2})\times \frac{t_{i}-t_{i-1}}{6}[J(t_{n}-t_{i-1})+4J(t_{n}-\frac{t_{i-1}+t_{i}}{2})+J(t_{n}-t_{i})]=t_{n}$$

Equation (6) is a system of linear equations with *n* unknown parameters, which can be written as Equation (7) [24]:

(7) Ax=B

A, x, and B in Equation (7) can be written as Equation (8) to Equation (10), respectively:

(8) $$A_{ij}=\{\begin{array}{lll} (t_{i}-t_{i-1})[J(t_{n}-t_{i-1})+4J(t_{n}-\frac{t_{i-1}+t_{i}}{2})+J(t_{n}-t_{i})], & if & j\leq i\\ 0, & if & j>i \end{array}$$

(9) $$x_{i}=G(\frac{t_{i-1}+t_{i}}{2})$$

(10) $$B=6t_{i}$$

for i,j∈{1,2,⋯,n}.

#### 2.2.4. Interconversion between Dynamic Modulus and Relaxation Modulus

**Figure 1:**
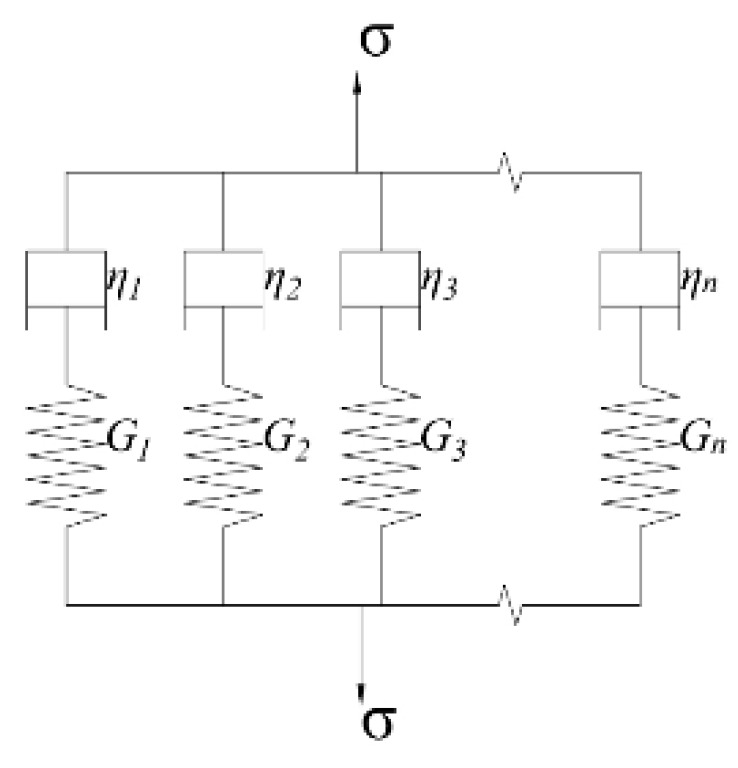
The generalized Maxwell model.

Some studies have pointed out that the variation of relaxation modulus of asphalt mixtures with loading time can be fitted by the generalized Maxwell model shown in Figure 1 and Equation (11) [18]. In addition, the dynamic modulus and relaxation modulus of asphalt mixtures shares the coefficients of the generalized Maxwell model; thus, the dynamic modulus of asphalt mixtures can be directly obtained by the fitting results of the relaxation modulus of asphalt mixtures. The calculated method of dynamic modulus $G^{*}$ is shown in Equation (12) [26,27]:

(11) $$G(t)=G_{e}+\sum_{i=1}^{n}G_{i}e^{-t/\rho_{i}}=G_{g}-\sum_{i=1}^{n}G_{i}(1-e^{-t/\rho_{i}})$$

(12) $$G^{*}(i\omega )=G_{e}+\sum_{i=1}^{n}G_{i}\frac{i\omega \rho_{i}}{1+i\omega \rho_{i}}=G_{g}-\sum_{i=1}^{n}G_{i}\frac{1}{1+i\omega \rho_{i}}$$

where $G_{e}$ is the equilibrium modulus; $G_{i}$ and $\rho_{i}$ are the relaxation strengths and relaxation time; i is the complex number; ω is the angular frequency.

The dynamic modulus $G^{*}$ of asphalt mixtures can be deducted by storage modulus $G^{\prime }$ and loss modulus $G^{\prime \prime }$, as shown in Equation (13). The phase angle δ can be calculated by Equation (14):

(13) $$G^{*}=G^{\prime }+iG^{\prime \prime }$$

(14) $$\delta =\frac{180\times G^{\prime }(\omega )}{\pi \times G^{\prime \prime }(\omega )}$$

According to the coefficients of the generalized Maxwell model, the storage modulus $G^{\prime }$ and the loss modulus $G^{\prime \prime }$ can be calculated by Equations (15) and (16), respectively [16,28,29]:

(15) $$G^{\prime }(\omega )=G_{e}+\sum_{i=1}^{m}\frac{\omega^{2}\rho_{i}^{2}G_{i}}{\omega^{2}\rho_{i}^{2}+1}=G_{g}-\sum_{i=1}^{m}\frac{G_{i}}{\omega^{2}\rho_{i}^{2}+1}$$

(16) $$G^{\prime \prime }(\omega )=\sum_{i=1}^{m}\frac{\omega \rho_{i}G_{i}}{\omega^{2}\rho_{i}^{2}+1}$$

## 3. Results and Discussion

### 3.1. Creep Characteristic Analysis of OGFC

#### 3.1.1. Creep Test Results of OGFC

**Figure 2:**
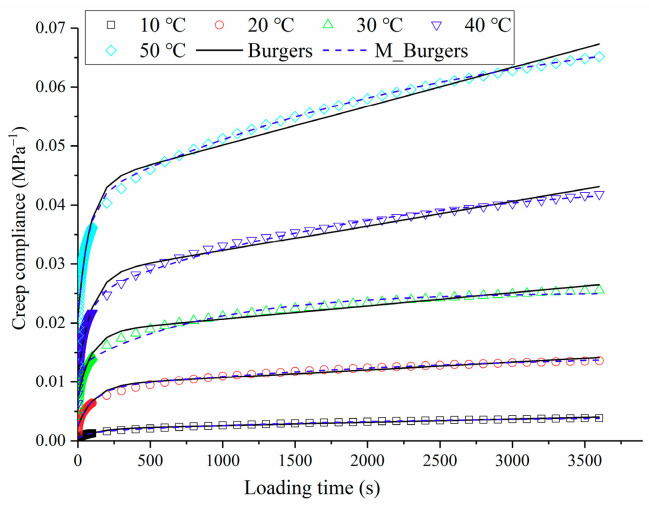
Creep compliance of OGFC at different temperatures and comparison of fitting results.

According to the uniaxial compression creep tests, the creep compliance of OGFC at 10 °C, 20 °C, 30 °C, 40 °C, and 50 °C was tested and calculated, and the average results are shown in Figure 2. As shown in Figure 2, at the initial stage of loading, the creep compliance of OGFC grows rapidly with the loading time; when the loading time is greater than 500 s, the creep compliance of OGFC grows gently, showing a nearly linear growth trend. It can also be found from Figure 2 that the creep compliance of OGFC increases significantly with the increase in test temperature. This is mainly due to the softening of rubber asphalt as binder after the temperature rises, which weakens the ability of creep resistance and increases the creep flexibility.

#### 3.1.2. Creep Characteristic Analysis Based on the Burgers Model and the Modified Burgers Model

**Figure 3:**
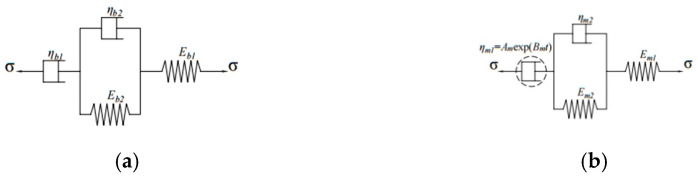
Creep compliance fitting model: (**a**) the Burgers model; (**b**) the modified Burgers model.

To further quantitatively analyze the influence of temperature on creep characteristics of OGFC, the Burgers model and modified Burgers model, shown in Figure 3 and Equations (17) and (18), were applied to fit the creep compliance curve [30,31,32,33]. The parameters of the two models are fitted by the programming algorithm in Excel software (Microsoft Corporation, Albuquerque, NM, USA) according to Equation (19). The fitting results are given in Table 3.

(17) $$J(t)=\frac{1}{E_{b1}}+\frac{t}{\eta_{b1}}+\frac{1}{E_{b2}}[1-\exp(-\frac{E_{b2}t}{\eta_{b2}})]$$

(18) $$J(t)=\frac{1}{E_{m1}}+\frac{1}{A_{m}B_{m}}(1-e^{-B_{m}t})+\frac{1}{E_{m2}}[1-\exp(-\frac{E_{m2}t}{\eta_{m2}})]$$

(19) $$\min\frac{1}{n}\sum_{i=1}^{n}{\left(\frac{J(t_{i})-J^{\prime }(t_{i})}{J(t_{i})}\right)}^{2}$$

where $E_{b1}$ and $E_{m1}$ are the instantaneous elastic modulus of asphalt mixtures, which can be employed to characterize the ability of asphalt mixture to resist instantaneous elastic deformation; $\eta_{b1}$ is the viscosity coefficient of asphalt mixtures, which can be utilized to assess the ability of asphalt mixture to resist the viscous resistance of asphalt mixture; $E_{b2}$ and $\eta_{b2}$ or $E_{m2}$ and $\eta_{m2}$ are used to reflect the viscoelastic properties of asphalt mixtures; $\tau_{b}=\eta_{b2}/E_{b2}$ is defined as the retardation time, which can be used to characterize the viscoelastic displacement growth rate of asphalt mixture; $A_{m}$ and $B_{m}$ are the parameters to be fitted to the modified Burgers model, and $A_{m}B_{m}$ can be applied to characterize the ability of asphalt mixtures to resist irreversible per-manent deformation; $J(t_{i})$ can be applied to characterize the ability of asphalt mixtures to resist irreversible permanent deformation; is the test value of creep compliance; $J^{\prime }(t_{i})$ is the prediction value of the fitting model for creep compliance; n is the number of test data. 

##### Instantaneous Elastic Deformation.

**Figure 4:**
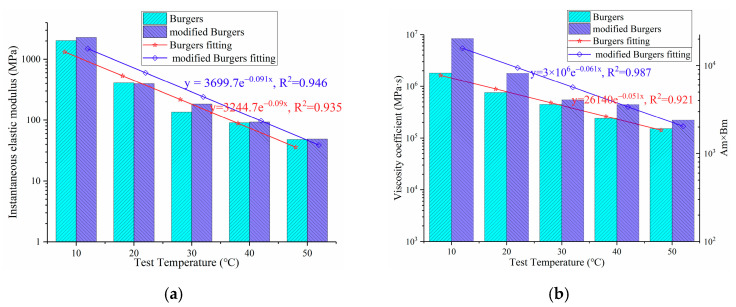
Variation of instantaneous elastic modulus and viscous resistance with test temperature: (**a**) Instantaneous elastic modulus; (**b**) irreversible permanent deformation.

According to Table 3, the variation of the instantaneous elastic modulus and viscosity coefficient of OGFC with test temperature is plotted, as shown in Figure 4. From the fitting results in Figure 4a, it can be seen that with the increase in test temperature, the instantaneous elastic modulus $E_{b1}$ and $E_{m1}$ of OGFC are decreasing, which indicates that the resistance to instantaneous elastic deformation of OGFC decreases with the increase in temperature. Moreover, the exponential function is employed to fit the variation of the instantaneous elastic modulus with temperature. From the fitting results of the exponential function, it can be found that the instantaneous elastic modulus of OGFC decreases as a negative exponential function with the increase in temperature. The rates of decline obtained by the Burgers model and modified Burgers model are very close.

##### Irreversible Permanent Deformation

Figure 4b shows the variation of the permanent deformation resistance of OGFC with the test temperatures. It can be seen from the figure that with the increase in the test temperature, $\eta_{b1}$ and $A_{m}B_{m}$ of OGFC decreases rapidly with a negative exponential function, which implies that the resistance to permanent deformation of OGFC decreases with the increase in test temperature. This is mainly attributed to the softening of the asphalt, which plays the role of bonding aggregate in the open graded asphalt mixture after the increase in temperature, so that the irrecoverable permanent deformation is more likely to occur in the asphalt mixtures. Furthermore, it can be found that the index $A_{m}B_{m}$ of permanent deformation resistance for the modified Burgers model is more sensitive to the change of temperature than the index $\eta_{b1}$ of the Burgers model. 

##### Viscoelastic Deformation

When the load time t tends to infinity, the modified Burgers model, as shown in Equation (18), can be written as Equation (20). From Equation (20), it can be found that the creep strain of asphalt mixtures is mainly composed of instantaneous elastic strain $\sigma_{0}/E_{m1}$, viscous strain $\sigma_{0}/(A_{m}B_{m})$ and viscoelastic strain $\sigma_{0}/E_{m2}$. Therefore, $E_{m2}$ can be used to characterize the viscoelastic deformation resistance of asphalt mixtures.

(20) $$J(t\to \infty )=\frac{\varepsilon_{c}}{\sigma_{0}}=\frac{1}{E_{m1}}+\frac{1}{A_{m}B_{m}}+\frac{1}{E_{m2}}$$

**Figure 5:**
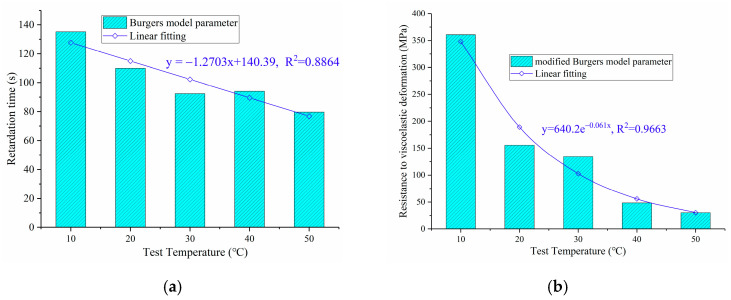
Variation of viscoelastic parameters of OGFC with test temperature: (**a**) $\tau_{b}$; (**b**) $E_{m2}$.

Figure 5 shows the variation of retardation time $\tau_{b}$ of the Burgers model and parameters $E_{m2}$ of the modified Burgers model with the test temperature. As shown in Figure 5a, with the increase in test temperature, the retardation time $\tau_{b}$ of OGFC presents a decreasing trend, which indicates that the growth rate of viscoelastic deformation is accelerated after the temperature increases, and accelerates the viscoelastic deformation of OGFC. As shown in Figure 5b, the parameter $E_{m2}$ of anti-viscoelastic deformation shows a negative exponential decreasing trend with the increase in test temperature, which implies that the anti-viscoelastic deformation ability of OGFC decreases rapidly with the increase in test temperature.

### 3.2. Relaxation Characteristic Analysis of OGFC

#### 3.2.1. Relaxation Modulus Calculation Results of OGFC

**Figure 6:**
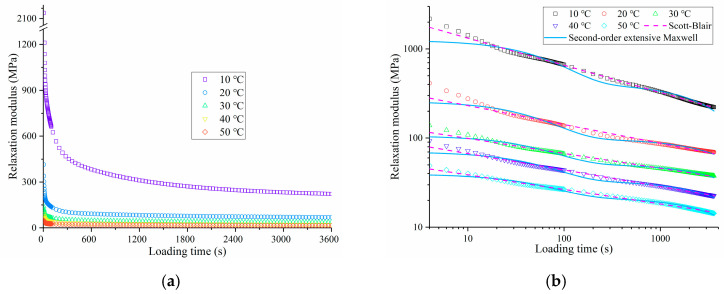
Relaxation modulus of OGFC at different temperatures: (**a**) Derivation results; (**b**) comparison of fitting results.

According to the relationship between the creep compliance and relaxation modulus of asphalt mixture in Equation (6), the relaxation modulus of OGFC at 10 °C, 20 °C, 30 °C, 40 °C, and 50 °C is calculated by using MATLAB software (MathWorks.Inc, Natick, MA, USA), as shown in Figure 6. It can be observed from Figure 6a that with the increase in loading time, the relaxation modulus of OGFC first decreases rapidly and then decreases slowly. 

In general, the attenuation process of the relaxation modulus of asphalt mixture can be divided into three stages. The first stage is the rapid decline stage, which is usually completed in tens of seconds, and the decline in the relaxation modulus is the greatest at this stage. The second stage is the turning stage of the decrease in the relaxation modulus. The decrease speed of the relaxation modulus gradually slows down, and the decrease range of the relaxation modulus is about 30% of the initial relaxation modulus. The third stage is the slow decline stage of the relaxation modulus, which indicates that the change of the relaxation modulus of asphalt mixtures has tended to be stable, and the decline of the relaxation modulus in this process is the smallest. At the end of the relaxation test, about 10% of the initial relaxation modulus remains.

Figure 6b shows the change in the relaxation modulus of OGFC with loading time in the double logarithm coordinate system. It can be found that the change in relaxation modulus with loading time can show good linearity in the double logarithm coordinate system. Moreover, with the increase in test temperature, the relaxation modulus of OGFC decreases rapidly, which means that the internal stress diffusion ability of OGFC rapidly deteriorates with the increase in temperature.

#### 3.2.2. Relaxation Characteristic Analysis Based on Second-Order Extensive Maxwell Model and Scott-Blair model

**Figure 7:**
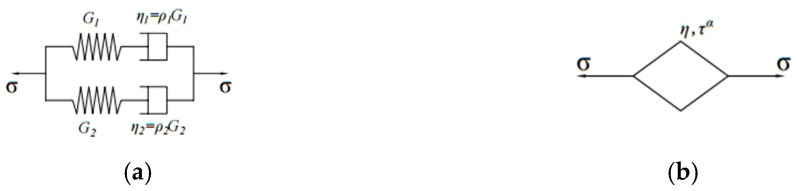
Relaxation modulus fitting model: (**a**) The second-order extensive Maxwell model; (**b**) the Scott-Blair model.

To further quantitatively analyze the effect of test temperature on relaxation characteristics of OGFC, the second-order extensive Maxwell model [34,35,36,37] and the Scott-Blair model [38], shown in Figure 7 and Equations (21) and (22), were employed to fit the relaxation modulus curve:

(21) $$G(t)=G_{1}e^{-t/\rho_{1}}+G_{2}e^{-t/\rho_{2}}$$

(22) $$G(t)=\eta \frac{t^{-\alpha }}{\int_{0}^{1-\alpha }e^{-\tau }\tau^{-\alpha }d\tau }$$

where $G_{1}$ and $G_{2}$ are the strengths to characterize the stress relaxation; $\rho_{1}$ and $\rho_{2}$ are the time employed to evaluate the release of internal stress in asphalt mixture; η and α are the viscous damping coefficient and fractional order, respectively; η can be utilized to assess the ability of asphalt mixtures to resist viscous deformation, and the value α is related to the rheological properties of asphalt mixtures; a greater fractional order α means that the rheological property of the asphalt mixture is closer to the fluid characteristics.

According to Equations (21) and (22), the parameters of the two models are calculated by programming algorithm in Excel. The calculated results are shown in Table 4, and the fitting results are compared with the derived relaxation modulus as shown in Figure 6b. From the correlation coefficients R^2^ in Table 4, it can be seen that the correlation coefficients are all above 0.9. The second-order generalized Maxwell model and Scott-Blair model can well describe the variation trend of relaxation modulus of OGFC with loading time at different temperatures. However, it is also obvious that Scott-Blair model is superior to the second-order generalized Maxwell model in describing the variation. The second-order generalized Maxwell model is poor in characterizing the variation of relaxation modulus of OGFC at short loading times. This is mainly because the spring element parameters of the Maxwell model mainly characterize the instantaneous change of the relaxation modulus of OGFC, while the viscosity coefficient parameters of the sticky pot element mainly represent the long-term relaxation modulus attenuation of OGFC, so the combination of the two is difficult to reflect the rapid decline of the relaxation modulus at the initial stage of loading.

From the fitting results of the second-order generalized Maxwell model in Table 4, it can be seen that the relaxation strength of OGFC decreases with the increase in the test temperature, and the relaxation ability of OGFC to the internal stress weakens when the temperature increases.

For the fitting results of the Scott-Blair model, it can be observed that the viscosity coefficient of OGFC decrease with the increase in test temperature, which indicates that the asphalt mixture changes from solid elastomer to fluid with the increase in temperature, its elastic properties and viscous resistance will decrease, and the anti-rutting deformation ability will weaken.

### 3.3. Dynamic Modulus Analysis of OGFC

#### 3.3.1. Dynamic Modulus Test Results of OGFC

**Figure 8:**
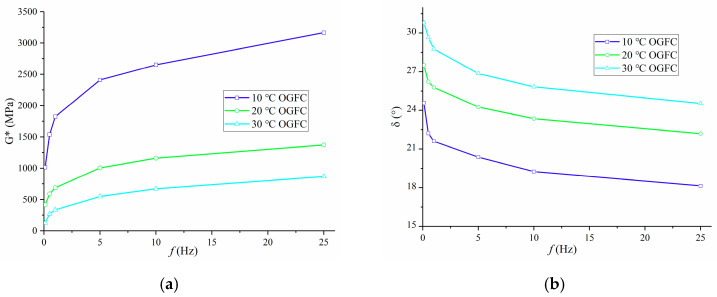
Variation of dynamic modulus and phase angle of OGFC with loading frequency at different temperature: (**a**) Dynamic modulus; (**b**) phase angle.

According to Equations (2) and (3), the dynamic modulus test results are calculated at 10 °C, 20 °C, and 30 °C, and the variation of dynamic modulus and phase angle of OGFC with loading frequency is plotted, as shown in Figure 8.

It can be seen from Figure 8a that with the increase in loading frequency, the dynamic modulus of OGFC increases continuously, and it is fast at first and then slow. Moreover, with the increase in test temperature, the dynamic modulus of OGFC decreases. At higher temperature or lower frequency, the asphalt mixture is softer, so the dynamic modulus of OGFC is lower, and the dynamic modulus is closer to the static modulus. When the test temperature or loading frequency is low, the asphalt mixture is hard, resulting in the larger dynamic modulus of the asphalt mixture, and the dynamic modulus at a lower temperature or higher frequency is closer to the glass modulus of the asphalt mixture.

From Figure 8b, it can be found that with the increase in loading frequency, the phase angle of OGFC decreases, at first rapidly and then slowly. Moreover, the phase angle of OGFC increases with the increase in test temperature. At higher temperature or lower loading frequency, the OGFC has a larger phase angle, indicating that it has higher viscosity.

#### 3.3.2. Dynamic Modulus Analysis Based on the Modified Sigmoid Model

**Figure 9:**
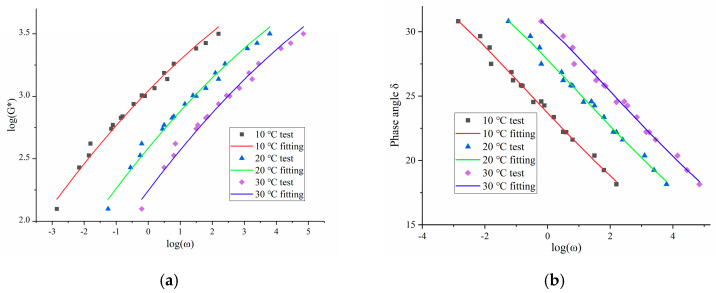
Variation of the dynamic modulus and phase angle of OGFC with the loading frequency: (**a**) Dynamic modulus; (**b**) phase angle.

The change in dynamic modulus of asphalt mixtures, as a typical viscoelastic material, with loading frequency meets the time-temperature equivalence principle of polymer materials, and the master curve of dynamic modulus of asphalt mixtures can be constructed by the shift factor, which can be used to study the development of dynamic modulus of asphalt mixtures in the range of higher or lower loading frequency. According to the time-temperature equivalence principle, the dynamic modulus master curves of OGFC are constructed in the double logarithmic coordinate axis based on the dynamic modulus curves at 10 °C, 20 °C and 30 °C, as shown in Figure 9. It can be seen from Figure 9 that at a wide range of loading frequencies (10^−3^–10^5^ Hz), the dynamic modulus of OGFC is still increasing with the increase in loading frequency.

In order to further analyze the variation of the dynamic modulus and phase angle of OGFC with the loading frequency and the influence of test temperature on the dynamic modulus and phase angle of OGFC, the modified Sigmoid model, as shown in Equations (23) and (24), is employed to fit the master curve of dynamic modulus and phase angle of OGFC [39,40]. The fitting results are shown in Figure 9 and Table 5.

(23) $$\log(G^{*})=\log(G_{\min})+\frac{\log(G_{\max})-\log(G_{\min})}{{\left(1+\lambda e^{\beta +\gamma \log\omega }\right)}^{\frac{1}{\lambda }}}$$

(24) $$\delta (\omega )=-\frac{\pi }{2}*\frac{[\log(G_{\max})-\log(G_{\min})]\gamma e^{\beta +\gamma \log\omega }}{{\left(1+\lambda e^{\beta +\gamma \log\omega }\right)}^{\frac{1}{\lambda }+1}}$$

where $G_{\min}$ and $G_{\max}$ are the static modulus and glassy modulus of OGFC, respectively, which are closely related to the gradation type, void ratio, test temperature and asphalt saturation of OGFC; $G_{\min}=\underset{\omega \to 0}{\lim}G*(\omega )$, $G_{\max}=\underset{\omega \to \infty }{\lim}G*(\omega )$; λ, β, and γ are the parameters to be fitted which can be applied to characterize the curve shape of the modified sigmoid model; ω is the angular frequency.

It can be seen from Figure 9 that the modified Sigmoid model can adequately characterize the changing trend of dynamic modulus and phase angle of OGFC at three test temperatures. From the variation of dynamic modulus of OGFC with loading frequency and the fitting results in Table 5, it can be found that under the higher or lower loading frequency, the dynamic modulus of OGFC tends to converge, converging to glass modulus at high frequency and static modulus at low frequency. Moreover, from the fitting results, it can be observed that the glass modulus $G_{\max}$ or static modulus $G_{\min}$, at the three test temperatures, tend to be almost the same. This indicates that OGFC has almost the same glass modulus $G_{\max}$ and static modulus $G_{\min}$ at different temperatures. This is mainly because the dynamic modulus at high temperature is equivalent to that at low loading frequency, and correspondingly the dynamic modulus at low temperature is equivalent to that at high frequency. The fitting results of Sigmoid model also show that the test temperature mainly affects parameter λ, but has little influence on parameters β and γ. With the increase in test temperature, the value of parameter λ increases.

### 3.4. Comparative Analysis of Dynamic Modulus of OGFC under Dynamic and Static Loads

From the fitting results of the second-order generalized Maxwell model, it can be seen that when the order of the generalized Maxwell model is small, it is poor to characterize the change of relaxation modulus in the short-term loading process. Therefore, in order to accurately establish the functional relationship of viscoelastic parameters of asphalt mixture under dynamic and static loading modes, the sixth order generalized Maxwell model was selected for subsequent calculation. Equation (10) is used to fit the relaxation modulus of OGFC at 10 °C, 20 °C, 30 °C, 40 °C, and 50 °C, and the fitting result is shown in Table 6. Substituting the data in Table 6 into Equation (12) to Equation (16), the dynamic modulus and phase angle of OGFC at 10 °C, 20 °C, and 30 °C are obtained.

**Figure 10:**
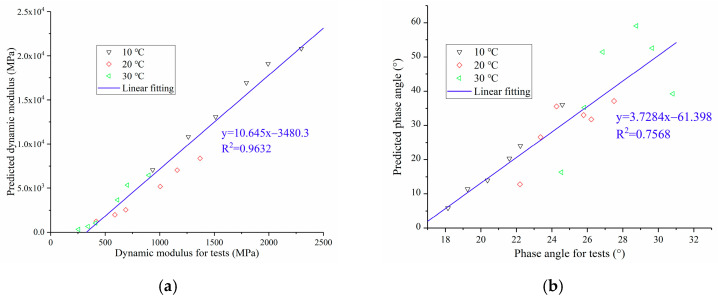
Comparison of the dynamic modulus and phase angle of OGFC obtained under the static and dynamic loading modes: (**a**) Dynamic modulus; (**b**) phase angle.

Figure 10 shows the comparison of the dynamic modulus and phase angle of OGFC obtained under the static and dynamic loads. As can be seen from Figure 10a, there is a great difference between the dynamic modulus obtained from the test and that derived from creep compliance, but there is a good linear correlation between them. Figure 10b shows that the phase angle obtained by the experiment and the derivation are scattered in the figure, which shows that the correlation between them is poor.

To better analyze the correlation of viscoelastic parameters of OGFC under dynamic and static loads, the linear correlation function is adopted to fit the scatter diagram. From the fitting results, it is found that the correlation coefficient of the dynamic modulus is 0.9632, while the correlation coefficient of the phase angle is only 0.7568, which shows that the dynamic modulus of OGFC obtained under the dynamic and static loads has a good linear correlation. The dynamic modulus obtained by the dynamic modulus test can be calculated by reasonably modifying that derived from creep compliance. The modified equation is shown in Figure 10a. However, the correlation of phase angle is poor, so it is difficult to establish the functional relationship between phase angle of OGFC under dynamic and static loads.

## 4. Conclusions

To analyze and compare the viscoelastic properties of OGFC under dynamic and static loads, uniaxial compression creep tests and the dynamic modulus tests were carried out to obtain the creep compliance, dynamic modulus, and phase angle. Furthermore, the viscoelastic theory and models were applied to study the effect of test temperature on viscoelastic properties of OGFC and to construct the functional relationship of viscoelasticity parameters of OGFC under dynamic and static loads. The following results can be summarized:

(1) With the increase in test temperature, the creep compliance of OGFC increases significantly, and the corresponding anti-instantaneous elastic deformation ability ($E_{b1}$ and $E_{m1}$), anti-irrecoverable permanent deformation ability ($\eta_{b1}$ and $A_{m}B_{m}$) and anti-viscoelastic deformation ability ($E_{m2}$) show negative exponential decline.
(2) With the increase in loading time, the relaxation modulus of OGFC decreased rapidly at first and then slowly; with the increase in test temperature, the relaxation modulus of OGFC decreased significantly. The Scott-Blair model can better describe the change in OGFC relaxation modulus with loading time than the second-order extensive Maxwell model, and the fractional order of the Scott-Blair model can adequately characterize the transition of OGFC from solid to fluid with the increase in temperature.
(3) With the increase in loading frequency, the dynamic modulus of OGFC increases and the phase angle decreases. The increase in test temperature will have an adverse effect on the dynamic modulus of OGFC. The fitting analysis results show that the dynamic modulus of OGFC at different temperatures almost converges to the same value at very high or very low loading frequency, converges to the glassy modulus around 10^5^ MPa at high frequency, and converges to the static modulus around 2 MPa at low frequency.
(4) The dynamic modulus derived from static creep compliance has a good linear correlation with that obtained by dynamic modulus tests, but the correlation of the phase angle is poor. The actual dynamic modulus of OGFC can be calculated and obtained by the linear function modified the dynamic modulus derived from creep compliance, which can provide a new way to obtain the parameters needed for the structural design of asphalt pavement.

## Figures and Tables

**Table 1:** Physical properties of rubber modified asphalt.

Properties	Standard	Results
Penetration (0.1 mm) at 25 °C	60–80	67.9
Ductility (cm) at 5 °C	≥20	26.8
Softening point T_R&B_ (°C)	≥65	76.4
Elastic recovery (%) at 25 °C	≥85	90.3
After rolling thin film oven test		
Mass loss (%)	≤±0.8	0.26
Residual penetration ratio (%) at 25 °C	≥60	81.5
Residual ductility (cm) at 5 °C	≥10	14.4

**Table 2:** Aggregate gradation of open-graded friction course (OGFC)-13.

Sieve Size (mm)	0.075	0.15	0.3	0.6	1.18	2.36	4.75	9.5	13.2	16
Percent passing (%)	4	5.5	7.5	9.5	12	16	21	70	95	100

**Table 3:** Fitting results of creep compliance of OGFC.

Fitting Model	Parameters	Test Temperature (°)				
		10	20	30	40	50
Burgers model	*E*_*b*1_ (MPa)	2031.74	409.42	136.06	90.30	47.97
	*η*_*b*1_ (MPa∙s)	1.81 × 10^6^	7.57 × 10^5^	4.47 × 10^5^	2.40 × 10^5^	1.52 × 10^5^
	*E*_*b*2_ (MPa)	646.61	143.43	90.37	58.69	44.06
	*η_b2_* (MPa∙s)	87,432.04	15,779.04	8351.56	5520.72	3510.45
	*τ_b_* (s)	135.2	110.0	92.4	94.1	79.7
	R^2^	0.9943	0.9912	0.9864	0.9887	0.9867
Modified Burgers model	*E_m1_* (MPa)	2290.66	397.43	184.10	92.88	48.88
	*A_m_*	71,157.57	15,954.89	72,040.56	4739.02	3328.47
	*B_m_*	0.0138	0.0101	0.0011	0.0156	0.0148
	*E*_*m*2_ (MPa)	360.79	155.54	134.28	48.47	30.28
	*η*_*m*2_ (MPa∙s)	6.84 × 10^5^	3.69 × 10^5^	3.25 × 10^3^	9.59 × 10^4^	8.10 × 10^4^
	R^2^	0.9983	0.9937	0.9963	0.9930	0.9902

**Table 4:** Fitting results of relaxation modulus of OGFC.

Fitting Model	Parameters	Test Temperature (°C)				
		10	20	30	40	50
Second-order extensive Maxwell model	*G*_1_ (MPa)	846.14	160.89	55.29	37.21	17.96
	*G*_2_ (MPa)	409.42	98.05	51.14	33.28	21.59
	*ρ*_1_ (s)	76.06	61.96	67.55	67.64	69.55
	*ρ*_2_ (s)	5076.08	9107.26	10,255.41	8208.08	7977.76
	R^2^	0.9180	0.9229	0.9616	0.9634	0.9731
Scott-Blair model	*η*	3483.14	442.90	164.96	117.90	63.53
	*α*	0.3048	0.2113	0.1678	0.1848	0.1634
	R^2^	0.9865	0.9518	0.9842	0.9901	0.9952

**Table 5:** Fitting results of the dynamic modulus of OGFC.

Test Temperature (°C)	Parameters				
	$\log(G_{\min})$	$\log(G_{\max})$	λ	β	γ
10	0.353	5.041	−0.746	−0.149	−0.585
20	0.297	5.041	−0.518	−0.149	−0.574
30	0.330	5.043	−0.356	−0.149	−0.582

**Table 6:** Fitting results of the generalized Maxwell model for the OGFC relaxation modulus.

Parameters	Test Temperature (°C)				
	10	20	30	40	50
*G_e_* (MPa)	23.26	41.88	30.86	12.03	7.12
*G*_1_ (MPa)	6573.63	6284.02	6284.02	6284.02	6283.98
*G*_2_ (MPa)	6573.63	1073.81	192.55	133.20	53.75
*G*_3_ (MPa)	6573.63	1073.81	192.55	133.20	53.75
*G*_4_ (MPa)	755.43	147.87	47.63	32.22	15.01
*G*_5_ (MPa)	424.01	67.33	24.56	16.50	7.87
*G*_6_ (MPa)	191.83	27.44	15.90	11.38	7.87
*G*_7_ (MPa)	191.83	27.44	4.91	10.24	7.10
R^2^	0.9953	0.9953	0.9985	0.9985	0.9992

## Data Availability

The data used to support the findings of this study are available from the corresponding author upon request.
